# On the use of two-time correlation functions for X-ray photon correlation spectroscopy data analysis

**DOI:** 10.1107/S1600576717000577

**Published:** 2017-02-17

**Authors:** Oier Bikondoa

**Affiliations:** a Department of Physics, University of Warwick, Gibbet Hill Road, Coventry CV4 7AL, UK; b XMaS, The UK–CRG Beamline, ESRF – The European Synchrotron, CS40220, F-38043 Grenoble Cedex 09, France

**Keywords:** two-time correlation functions, X-ray photon correlation spectroscopy, data analysis

## Abstract

Multi-time correlation functions are especially well suited to study non-equilibrium processes. In particular, two-time correlation functions are widely used in X-ray photon correlation experiments on systems out of equilibrium. One-time correlations are often extracted from two-time correlation functions at different sample ages. However, this way of analysing two-time correlation functions is not unique. Here, two methods to analyse two-time correlation functions are scrutinized, and three illustrative examples are used to discuss the implications for the evaluation of the correlation times and functional shape of the correlations.

## Abstract 2

Two ways to analyse two-time correlation functions and the implications for the evaluation of the correlation times and functional shape of the correlations for equilibrium and non-equilibrium systems are discussed.

## 1. Introduction   

X-ray photon correlation spectroscopy (XPCS), the equivalent of dynamic light scattering using X-rays instead of visible light, is a powerful technique to study the dynamics of soft and hard condensed matter (Grübel *et al.*, 2008 ▸; Sutton, 2008 ▸; Gutt & Sprung, 2015 ▸; Madsen *et al.*, 2015 ▸; Bikondoa, 2016 ▸). XPCS allows one to probe the dynamics of fluctuations on short length scales (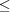
100 nm) and long time scales (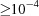
 s) (Malik *et al.*, 1998 ▸; Madsen *et al.*, 2010 ▸). Information about the dynamics is obtained by studying the time correlation of the intensity scattered by a system in a dynamic regime when illuminated with coherent light. Under coherent illumination, the far-field pattern of light scattered by a sample shows a grainy intensity distribution called speckle (Sutton *et al.*, 1991 ▸). The intermediate scattering function of the sample, 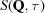, is obtained from the normalized intensity autocorrelation of the speckles, 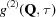, through the Siegert relation:^1^

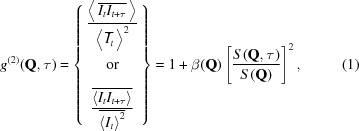
where 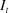 and 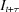 are the intensities at times *t* and 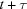 and at momentum transfer 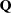. τ is a delay time. The superscript (2) marks that the intensity autocorrelation is a second-order correlation on the electric field. The bar 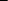 indicates an ensemble average over wavevectors with equivalent 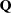 momentum transfer value and for which it is expected that the correlations are statistically equivalent. The brackets 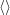 denote a time average.^2^ 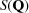 is the static structure factor. The optical contrast 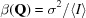 is a factor that is used to account for the degree of spatial coherence of the incident radiation and is given by the variance of the intensity (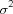) divided by its mean value (Madsen *et al.*, 2010 ▸). The calculation of 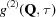 in equation (1) assumes that a time average can be performed over the entire measurement (Goodman, 1985 ▸). This assumption is valid for systems in equilibrium because for such systems the autocorrelation 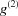 depends only on 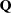 and the time difference or delay time τ between measurements. That is, 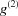 is time-shift invariant and does not depend on the specific time when the measurement was made (observation time). 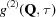 is a one-time correlation function (1-TCF).

For non-equilibrium systems (*i.e.* for systems with average properties changing with time) the time average in equation (1) is not suitable because the dynamics are evolving and may strongly depend on the observation time. For those systems the evolution of the correlations can still be captured by using a more general expression than equation (1), namely a two-time correlation function (2-TCF) (Brown *et al.*, 1997 ▸): 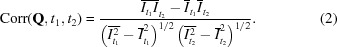
Corr is the autocovariance of the intensity normalized by its standard deviation. Different correlation functions are also used (Sutton *et al.*, 2003 ▸): 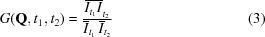
or 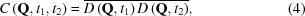
where 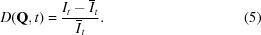
For random Gaussian fluctuations the standard deviation equals the average intensity (Brown *et al.*, 1997 ▸; Loudon, 1983 ▸). Therefore, 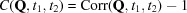 and the different correlation functions [equations (2), (3) and (4)] are equivalent.

The use of 2-TCFs for XPCS was introduced, to our knowledge, by Brown *et al.* (1997 ▸), who studied the time correlations in the intensity scattered by a phase ordering system using numerical simulations. The 2-TCF is generally represented as a two-dimensional graph of the value of 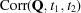, 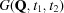 or 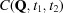 for a fixed 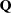, with axes 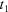 and 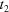 (see §2.2). Brown *et al.* (1997 ▸) introduced an alternative coordinate system, which has subsequently been widely used in the XPCS literature (Malik *et al.*, 1998 ▸; Brown *et al.*, 1999 ▸; Livet *et al.*, 2001 ▸; Sutton *et al.*, 2003 ▸; Fluerasu *et al.*, 2005 ▸; Ludwig *et al.*, 2005 ▸; Fluerasu *et al.*, 2007 ▸; Müller *et al.*, 2011 ▸; Orsi *et al.*, 2010 ▸, 2012 ▸; Chushkin *et al.*, 2012 ▸; Livet & Sutton, 2012 ▸; Bikondoa *et al.*, 2012 ▸; Ruta *et al.*, 2012 ▸). Using this alternative coordinate system, approximated 1-TCFs are often extracted from the 2-TCF at different sample ages or observation times. We show below that in some cases employing such a coordinate system to extract approximate 1-TCFs may pose interpretation problems. For such cases, we put forward another coordinate system to extract the 1-TCFs and propose a clearer graphical representation of the 2-TCFs.

This article is organized as follows: in §2 we describe the calculation of the autocorrelation (§2.1) and the two-time correlation function (§2.2) in discrete form. The extraction of 1-TCFs from the 2-TCF using different coordinate systems is examined in §3. Some properties of the 2-TCF for stationary and non-stationary systems, such as the time reversal symmetry, the functional shape and decay times, are analysed in §4. Model examples of 2-TCFs that reflect the differences between the analysis done using one coordinate system or another are presented in §5. A discussion about the coordinate system that should be used for different cases follows in §6. In §7 we propose, in our view, a clearer graphical representation of the 2-TCFs. A summary (§8) and an appendix that introduces a geometric interpretation of the multi-time correlation functions in terms of metric spaces (Appendix *A* [App appa]) close the article.

## 2. Calculation of the correlation functions   

### 2.1. Autocorrelation function   

**Figure 1:**
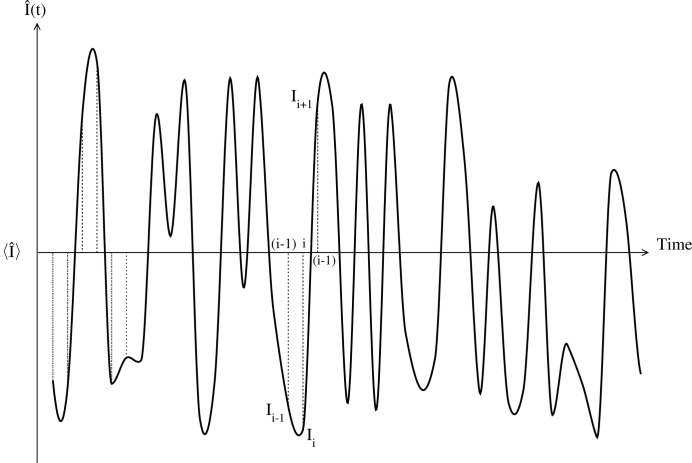
Generic sample function of a random process 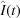 fluctuating in time around its average value 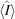. The time axis is divided into discrete time intervals.

We start by constructing the one-time correlation function (autocorrelation) for a generic set of data. The time autocorrelation function of a process 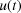 is defined by (Goodman, 1985 ▸) 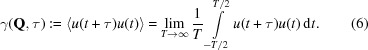
Let us consider that in a experiment we measure intensity fluctuations in time and at points 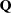 in reciprocal space.^3^ We can express a set of measured intensity fluctuations at different times as an *n*-tuple:
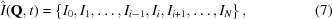
where the terms 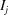 are the intensity fluctuations measured at times 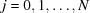 and are real numbers. A generic sample function is displayed in Fig. 1 ▸. The autocorrelation function is defined, in discrete form, by 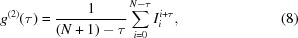
where 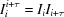. The terms of equation (8) corresponding to the different delay times (τ) are shown in Table 1 ▸. There are 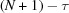 terms for a given delay time. For 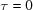, (*N* + 1) terms are averaged, for 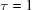, 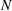 terms and so on.

### 2.2. Two-time correlation function   

If the process is not stationary, the statistical properties of the fluctuations will evolve over time. Thus, the summation and averaging that is done over the measurement time in equation (8) is not appropriate. A more general expression, namely a two-time correlation function (2-TCF), is obtained if the average in equation (8) is not performed. The 2-TCF is very useful to analyse the dynamics of non-equilibrium systems (Sutton *et al.*, 2003 ▸). The temporal fluctuations and the variance of the 2-TCF are also used to investigate dynamical heterogeneities in glassy systems through the analysis of higher-order correlations and multi-point dynamic susceptibilities [see Orsi *et al.* (2012 ▸) and Conrad *et al.* (2015 ▸) for recent XPCS work and references therein for details on the use of higher-order correlations to study dynamical heterogeneities].

The 2-TCF 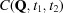 is obtained by calculating the Car­tesian product of 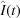 [equation (7)] with itself (see also Appendix *A* [App appa] for the calculation of the 2-TCF using the terminology of metric spaces) and ensemble averaging over equivalent 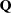 momentum transfer vectors or pixels, when using a two-dimensional detector (Lumma *et al.*, 2000 ▸): 
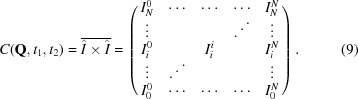
The 2-TCF is a symmetric matrix by construction.^4^ That is, the 2-TCF is symmetric upon index swapping, *i.e.* 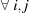: 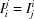, or, equivalently, 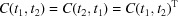, where T denotes the transpose operation. The time difference between two elements of the 2-TCF matrix is obtained using an 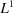-metric (also known as Manhattan, city-block or taxicab metric; Deza & Deza, 2014 ▸); the temporal distance (in units of scaled time) between two points 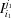 and 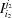 is obtained from the sum of the absolute value of the differences between their row and column indexes: 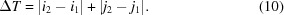
The elements with equal row and column indexes (terms of the form 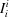) are ‘equal-time’ terms. For a generic equal-time term 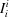, if the start of the experiment is taken as 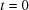 for 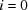, the time elapsed from the start of the experiment is 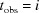. We shall call this elapsed time the observation time 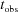. The temporal distances 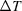 [equation (10)] can be converted into absolute time differences by multiplying them by the time step 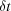. The autocorrelation function equation (8) is obtained by averaging the terms along lines parallel to the 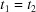 diagonal.

## 3. Analysis of two-time correlation functions using different time coordinate systems   

The evolution of the correlation functions is often quantified by selecting slices of the 2-TCFs at different observation times. These slices can be taken in different ways, using different coordinate systems. We discuss here the two most common procedures in the literature. Before proceeding, we should note, however, that other time variables such as 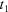, 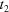 could also be used to define an ‘observation time’.

### 3.1. Conventional coordinate system   

Starting from an equal-time term 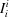 in the 2-TCF matrix [equation (9)], 1-TCFs at different observation times can be extracted by taking the delay time along rows or columns in equation (9), *i.e*. lines with 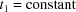 or 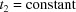. The terms in these 1-TCFs have the form 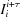 and the delay time τ is given by 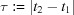. This way of extracting 1-TCFs arises directly from equation (8), removing the summation over *i* and the normalization factor that takes into account the number of terms summed for each delay time. We shall call this coordinate system the ‘conventional coordinate system’ (CCS), although we note that this coordinate system is rarely used in the XPCS literature to analyse 2-TCFs. The terms at different delay times are shown in Table 2 ▸. The autocorrelation [equation (8)] is obtained from the 2-TCF by averaging the terms at equal delay times τ of all the CCS-1-TCFs extracted at different observation times.

### 3.2. Alternative coordinate system   

**Figure 2:**
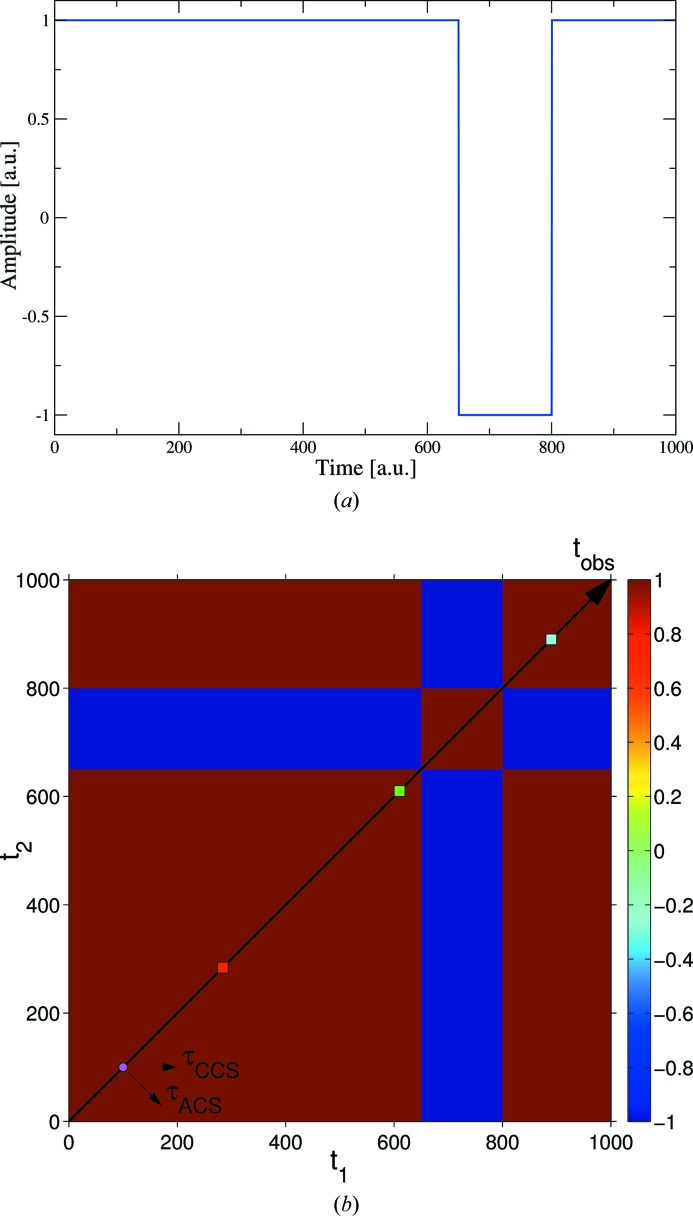
(*a*) Profile of the intensity. (*b*) Corresponding 2-TCF. The arrow running from the bottom left to the top right corner denotes the observation time. The delay time directions according to the CCS and ACS for an observation time 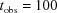 are indicated by the small arrows close to the bottom left corner. The small squares along the observation time correspond to observation times 284 (red), 610 (green) and 895 (cyan). The colour bar scale shows the degree of correlation.

An ‘alternative coordinate system’ (ACS) was introduced by Brown *et al.* (1997 ▸) and has become the customary coordinate system in XPCS to analyse the 2-TCFs to extract 1-TCFs from them at different sample ages. In this ACS, the sample age is taken along the 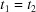 diagonal and defined as 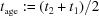. The delay time magnitude is the same as in the CCS system (*i.e.* 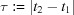), but starting from an equal term 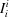, the delay time direction is taken along lines perpendicular to the 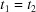 diagonal (see Fig. 2*b* ▸). Cuts of the 2-TCF along these perpendicular lines are 1-TCFs and are defined as ‘constant sample age’ cuts. These 1-TCFs are symmetric by construction around the 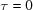 value. The terms at different delay times that are obtained for a given observation time 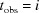, following the definition of Brown *et al.* (1997 ▸), are schematically shown in equation (11) (bold elements) and displayed in Table 2 ▸. Using the ACS, the autocorrelation function [equation (8)] is also obtained by averaging the terms at different delay times. 
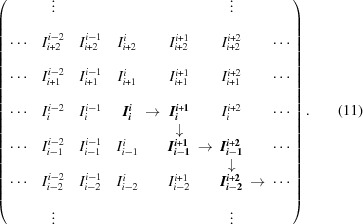



### 3.3. Differences between the CCS and ACS one-time correlation functions   

**Figure 3:**
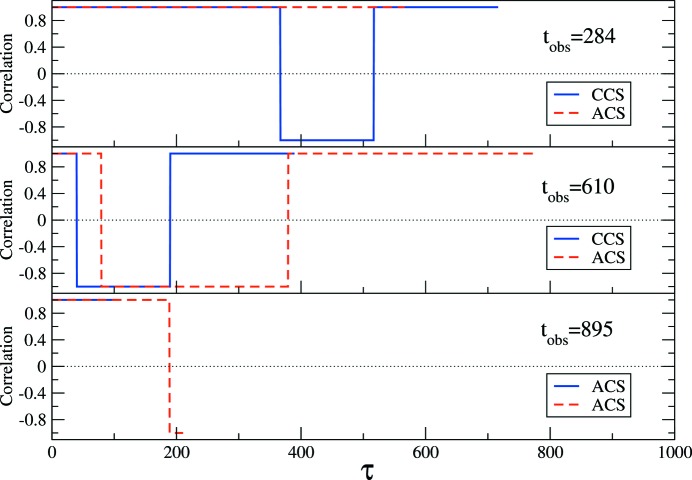
1-TCFs of the step function plotted in Fig. 2 ▸(*a*), extracted from its corresponding 2-TCF (Fig. 2 ▸ *b*) at observation times 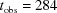, 610, 895, using the CCS (solid blue line) or ACS (dashed red line).

There are essential differences between the 1-TCFs that are extracted using the conventional or the alternative coordinate systems. The CCS-1-TCF that passes through an equal-time term 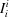 has terms of the form 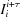, while the ACS-1-TCF comprises terms of the form 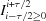 (τ even) or 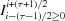 (τ odd) (see Table 2 ▸). Thus, for a 1-TCF extracted for an observation time^5^ 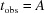 we can note two differences:

(1) The terms of the CCS-1-TCF are of the form 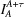 and thus are directly related to the intensity measured at time 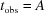. On the other hand, in a ‘constant sample age’ cut of the 2-TCF using the ACS, the terms have the form 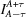: terms obtained from the multiplication of intensities measured at times before and after 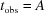 are mixed (see terms in Table 2 ▸). The ACS-1-TCF correlates terms that are equidistant (in time) from the observation time 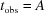, but these terms are not directly related to the intensity at the observation time 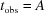, except for the terms 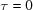.

(2) The number of terms of the 1-TCFs extracted for 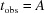 using the CCS or the ACS are different. For the CCS, the longest 1-TCF that can be extracted is at 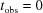 (beginning of the experiment). In contrast, using the ACS, the longest delay times accessible are for observation times 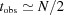, while the delay times accessible close to the start or end of the experiment are much shorter (see Fig. 3).

The first difference arises from the difficulty of defining precisely a ‘constant sample age’ in the case of time correlation functions. Time correlation functions are constructed by multiplying terms measured at different times. What happens at a certain time is related to another event at another time. ‘Constant sample age’ is then ambiguous and may be interpreted or defined in different ways. On one hand, a ‘constant’ age may be considered what happens to the state of the sample at time 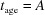 when it is related to its state at other times. This interpretation would be in line with the analysis done using the CCS. Or it may be interpreted as what happens when events that occur before and after 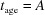 and at an equidistant time delay 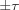 are compared, which would correspond to the ACS analysis.

The second difference is just a consequence of the choice of the coordinate system. However, the direction in which the delay time is taken it is extremely important when performing quantitative analysis, because, for non-equilibrium systems, the relaxation times obtained from CCS- or ACS-1-TCFs will be different (see §5.3).

## 4. 2-TCFs for stationary and non-stationary systems   

In stationary systems (strictly speaking, for wide-sense stationary systems; see Goodman, 1985 ▸), the 1-TCFs depend only on the time difference, not on the observation time. Therefore, the 2-TCF of a wide-sense stationary system is a Toeplitz matrix, *i.e.* the following relationship between the terms of the 2-TCF holds: 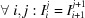. In addition, as the 2-TCF is symmetric around the 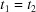 diagonal, then 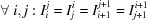. The 2-TCF [equation (9)] of a wide-sense stationary process thus has the form 
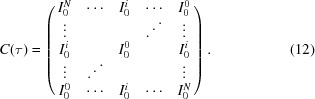



For wide-sense stationary systems, the CCS- and ACS- 1-TCFs are therefore equivalent, except for the number of terms for each 1-TCF. The time symmetry is also maintained for stationary processes. The autocorrelation function of a real (*i.e*. not complex) stationary process has the following property (Goodman, 1985 ▸): 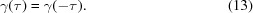
This property is fulfilled for the CCS- and ACS-1-TCFs of stationary processes because 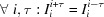 (CCS) and 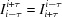 (ACS). However, in non-stationary processes equation (13) does not necessarily hold (*i.e.* the time symmetry is broken). Besides, the 1-TCFs will generally depend on the observation time 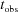 and the delay time τ. The breakdown of the time symmetry is well reflected in the CCS coordinate system: non-stationary processes yield asymmetric CCS-1-TCFs. However, even for non-stationary processes, equation (13) is fulfilled for the ACS-1-TCFs because they are symmetric by construction.

## 5. Examples   

It is illustrative to compare the CCS-1-TCF and ACS-1-TCF for some model, extreme cases. Three examples are presented below: the first two examples are based on simple mathematical functions and the third is based on the integration of a partial differential equation that has been proposed to describe the evolution of a semiconductor surface upon ion beam sputtering (Castro *et al.*, 2005 ▸). These examples have been chosen not for their physical relevance but because they reflect well some of the issues that arise when using different coordinate systems to extract 1-TCFs from 2-TCFs. The first example (§5.1: intensity following a step function) manifests that the ACS convention breaks the causality by mixing terms before and after an event has happened. In the second example (§[Sec sec5.2]5.2: sinusoidal intensity variation), the ACS-1-TCFs give skewed correlation functions and the skewness depends on the observation time. The third example (§[Sec sec5.3]5.3: 2-TCF of self-organized nanostructure formation dynamics on a surface due to sputtering) shows that, for an ageing system, the choice of the delay time direction has a direct effect on the correlation times and can also affect the functional shape of the correlation function.

In all the examples, we assume that the functions used in the calculations are representative of the dynamics of the system, *i.e.* that proper corrections, normalization and ensemble averaging of the raw data have been performed, as would indeed be required in a real DLS or XPCS experiment (for details, see *e.g.* Chu, 2007 ▸; Wong & Wiltzius, 1993 ▸; Madsen *et al.*, 2010 ▸; Madsen *et al.*, 2015 ▸).

### 5.1. Correlation function of a step intensity function   

We consider a dynamical system yielding intensity fluctuations in the scattered signal that can be described by a step function: 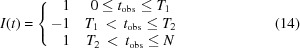



The signal profile is plotted in Fig. 2 ▸(*a*): the signal jumps from 1 to −1 at 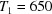 and goes back to 1 at 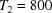. The corresponding 2-TCF is shown in Fig. 2 ▸(*b*). CCS- and ACS- 1-TCFs extracted at observation times 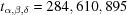 are displayed in Fig. 3 ▸.

We observe that the CCS-1-TCFs are correlated from the observation time 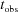 and 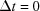 until the end of the period (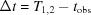) and that in the following period they are anticorrelated (*i.e.* *C* = −1). However, the ACS-1-TCFs show correlation from the observation time until delay times that are twice those of the CCS-1-TCFs. This is due to the different delay time directions and the Manhattan geometry of the 2-TCF; the CCS-1-TCF follows a line while the ACS-1-TCFs follow a staircase trajectory [see equation (11)]. For this reason, the ACS-1-TCFs change sign at different delay times than the CCS-1-TCFs. The ACS-1-TCFs are correlated for delay times that are longer than the difference between the observation time and the switching of the intensity which, physically, is inconsistent.

### 5.2. Correlation function of a periodically oscillating intensity   

**Figure 4:**
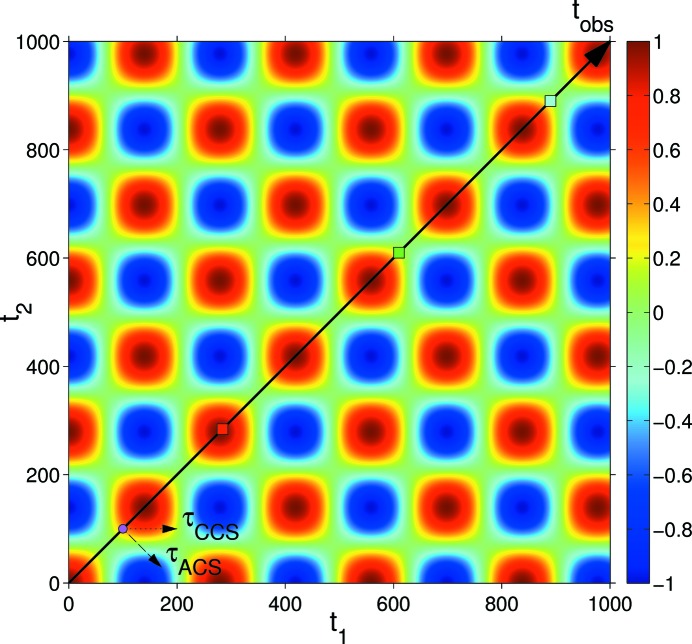
2-TCF of a sinusoidally oscillating intensity (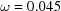). The arrow running from the bottom left to the top right corner denotes the observation time. The delay time directions according to the CCS and ACS for an observation time 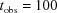 are indicated by the small arrows close to the bottom left corner. The small squares along the observation time correspond to observation times 284 (red), 610 (green) and 895 (cyan). The colour bar scale shows the degree of correlation.

**Figure 5:**
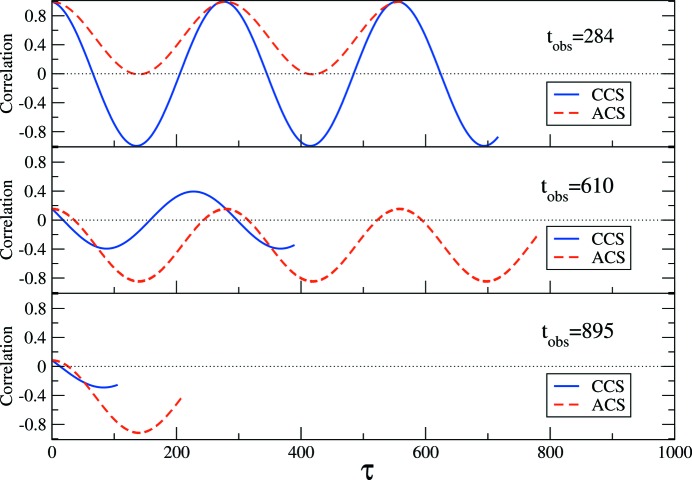
1-TCFs extracted from the 2-TCF of Fig. 4 ▸ at observation times 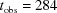, 610, 895, using the CCS (solid blue line) or ACS (dashed red line).

Let us consider a system where, because of its dynamics, the scattered intensity fluctuates around a constant mean value in a sinusoidal way with angular frequency ω. The intensity fluctuation can be represented as 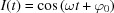, where 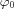 is the phase at time 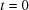. For simplicity, we take 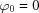 in the following. For such a signal, comparing the signal at time *t* with itself for different delay times τ, it is expected that the correlation of the signal should vary periodically from positive to negative. The autocorrelation, as calculated using equation (6), is 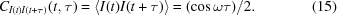
The 2-TCF is shown in Fig. 4 ▸. The terms of the 2-TCF have the form 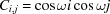. The 1-TCFs that are extracted from the 2-TCF following the CCS or the ACS convention have the following form: 
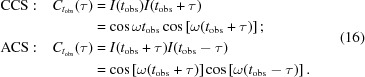
The 1-TCFs extracted at 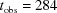, 610, 895 with 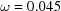 are shown in Fig. 5 ▸. Using the CCS, the (*a priori*) expected behaviour is reflected in the 1-TCFs, namely, the correlation oscillates periodically from positive values to negative ones and *vice versa*. The amplitude of the oscillations of a 1-TCF at time 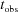 is determined by the value of 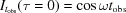. The amplitudes of the CCS-1-TCFs are symmetric around zero.

However, the 1-TCFs obtained with the ACS have a different behaviour: they may sometimes be always positive or negative. The behaviour of the 1-TCF extracted at 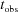 following the ACS convention can be determined more easily by rewriting equation (16) as 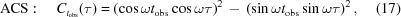
where we have used trigonometric identities to rewrite the expression. The two terms in equation (17) are positive and the amplitude of the ACS-1-TCFs will oscillate between 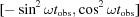. Thus, if 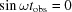 (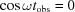), 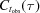 will always be positive (negative). This can be observed in the top panel of Fig. 5 (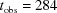): the correlation is always positive. For other 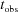 values, the amplitude variation of the correlations is not symmetrical and will be skewed to positive or negative values unless 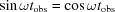.

### 5.3. Surface evolution under ion beam sputtering   

**Figure 6:**
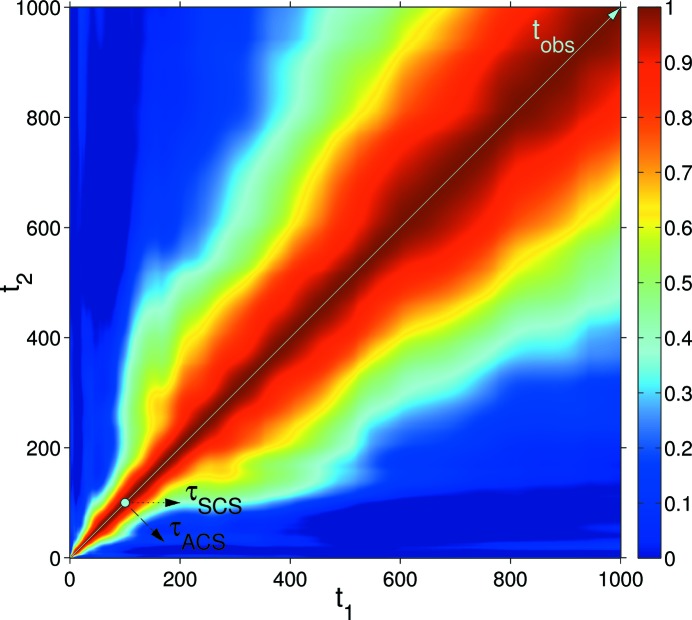
Contour plot of a 2-TCF for a model system that describes the evolution of a semiconductor surface under ion bombardment (Bikondoa *et al.*, 2012 ▸). The directions of the delay time (τ) for the CCS and ACS are indicated. The colour bar scale shows the degree of correlation.

**Figure 7:**
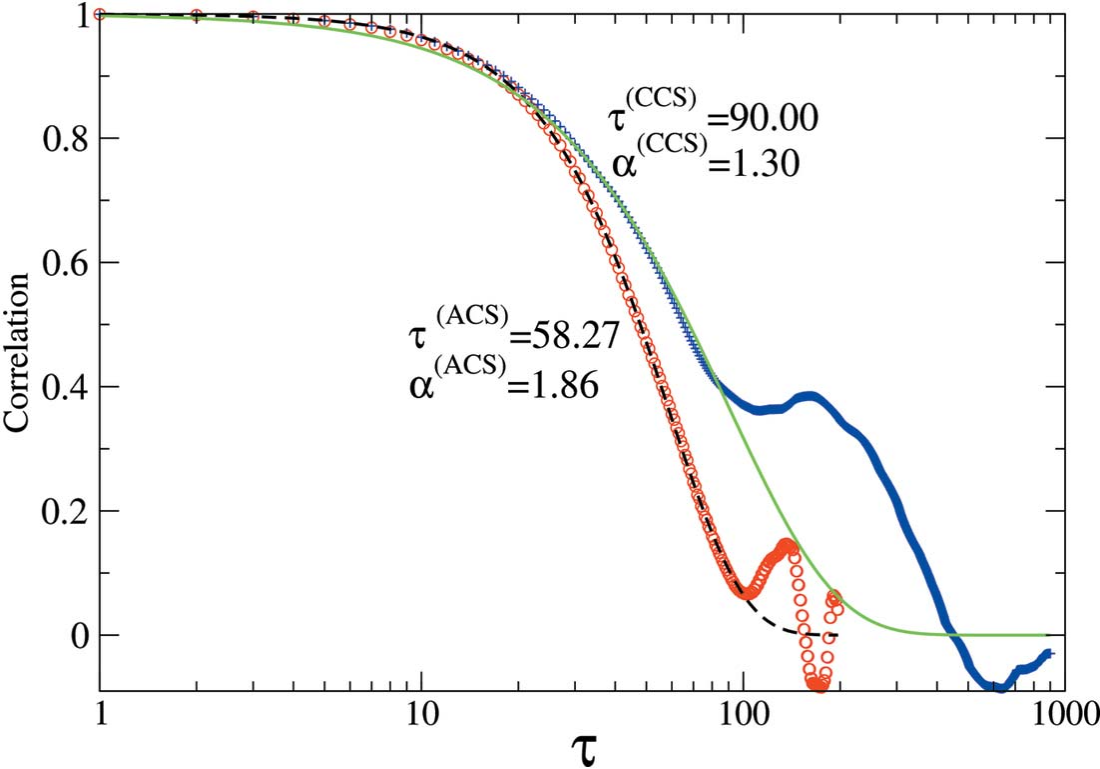
Example of one-time correlation functions at 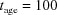 extracted from the 2-TCF of Fig. 6 ▸ using the convention of Brown *et al.* (1997 ▸) (open red circles) and the new convention proposed here (blue crosses). The dashed (black) and solid (green) lines have been obtained by fitting the one-time correlation data with the function 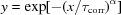, where τ is the correlation time and α the Kolhrausch–Williams–Watts exponent (Madsen *et al.*, 2010 ▸). For the fits, only the data up to 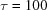 have been considered.

Ion beam sputtered surfaces are non-equilibrium systems that show ageing (Bikondoa *et al.*, 2013 ▸). One theoretical approach to describe the temporal evolution and dynamics of such systems is the continuum theory, which uses partial differential equations to describe the evolution of the surface height (Muñoz-García *et al.*, 2009 ▸). Fig. 6 ▸ displays the 2-TCF obtained from numerical simulations integrating an equation that describes the evolution of semiconductor surfaces under ion bombardment [for more details on such systems and the calculation of the 2-TCF, see Bikondoa *et al.* (2012 ▸), and references therein]. In Fig. 7 ▸, we have extracted CCS and ACS 1-TCFs from Fig. 6 ▸, for 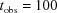. In the case of the ACS-1-TCF (open circles), only delay values up to 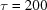 are accessible. For the CCS-1-TCF (crosses), a delay time up to 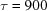 can be extracted. The two 1-TCFs have been fitted using a stretched exponential 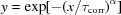, where 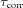 is the correlation time and α is the Kohlrausch–Williams–Watts exponent (Pecora, 2008 ▸). The value of the exponent α depends on the microscopic nature of the dynamics (Madsen *et al.*, 2010 ▸). Only the values in the 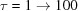 range have been used for the fit. This example shows (see values in Fig. 7 ▸) that, for a non-equilibrium system, there may be important differences in both the correlation times and the α exponents that are obtained using one convention or the other. Such differences may be extremely important when interpreting results from 2-TCFs and modelling the underlying dynamics (see the *Discussion* [Sec sec6]).

## 6. Discussion   

Which coordinate system should be used when analysing 2-TCFs and extracting 1-TCFs? *A priori*, either of the two coordinate systems can be used, provided, obviously, the comparison with theoretical models is done accordingly. This is the procedure followed in the pioneering work of Brown *et al.* (1997 ▸), in which computer simulations are used to study the statistical properties of speckles arising from the scattering of coherent radiation by a phase-ordering system. Theoretical models for such systems predict two-point, two-time correlation functions of the order parameter 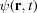 (*i.e.* the scalar field that describes the inhomogeneity of the system). The structure factor, which is obtained by averaging the scattered intensity over an ensemble of initial conditions, is related to the modulus square of the Fourier transform of the order parameter. Brown *et al.* (1997 ▸) analysed the intensity 2-TCFs using the ACS-1-TCF reference system, and the comparison with theoretical models and the scaling functions that they predict was done taking into account the ACS modified coordinates. The same procedure has been used in subsequent theoretical and experimental work on related or similar systems (Brown *et al.*, 1997 ▸; Livet *et al.*, 2001 ▸; Fluerasu *et al.*, 2005 ▸). However, for most cases the interpretation of the CCS-1-TCFs is more straightforward because its calculation is in line with the usual way of calculating time correlation functions in statistical mechanics: a function of the state of the system at an initial time is multiplied by the value of the function at another, later time *t*; the autocorrelation function is defined as the ensemble average of that product (Zwanzig, 1965 ▸). CCS-1-TCFs are also in accordance with the use of dynamic correlations and response functions to analyse how a function of the system responds to a perturbation applied at a certain time 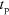 [for an account of the relationships between response and correlation functions, see Chaikin & Lubensky (1995 ▸) or Cugliandolo *et al.* (1994 ▸)]. The time symmetry is broken by applying an external field or force at time 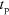. The response function will be nonzero only for 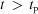. To account for this, a step function dependence on the time is often included in the definition of the response function. As shown in the example of §5.1, causality between terms of the correlation function is not retained for the ACS-1-TCFs and events that happen before and after the perturbation has occurred (*i.e.* 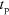) are then mixed. Thus, for the analysis of such systems, the use of CCS-1-TCFs seems to be better suited. The same applies for quenched systems: ACS-1-TCFs would mix events prior and subsequent to the quenching. This could be avoided if the ACS analysis is restricted to areas in the TCF that are not crossed by any of the ‘events’. That would entail remaining inside a single square area (either red or blue, in Fig. 2 ▸) without crossing the boundary to another area.

Extracting CCS-1-TCFs from the 2-TCFs is an equivalent procedure to that employed to analyse the contact dynamics on granular piles subjected to weak vibrations using multi-speckle diffusive wave spectroscopy (MDWS) (Kabla & Debrégeas, 2004 ▸). A waiting time is used to account for the number of vibrations the system has suffered before the measurement starts and a delay time for the number of vibrations after the waiting time. The waiting time is equivalent to the ‘observation time’ (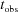) that has been defined above. The slow dynamics in glasses studied with dynamic light scattering have also been analysed in a similar manner, using a waiting time or sample age (Cipelletti *et al.*, 2000 ▸). In these two studies, the 2-TCF is not explicitly employed. We note here that theories of non-equilibrium phenomena are generally expressed in terms of correlations that follow the CCS formulation (see *e.g.* Van Vliet, 2008 ▸; Berthier *et al.*, 2011 ▸).

Ageing phenomena in glasses and other out-of-equilibrium systems have been extensively studied with XPCS using 2-TCFs and ACS-1-TCFs (Madsen *et al.*, 2015 ▸; Bikondoa, 2016 ▸). Thus, to compare quantitative values extracted from ACS-1-TCFs with values obtained using other experimental techniques (*e.g.* MDWS or DLS) or theoretical predictions, it may be necessary to perform a coordinate change to analyse the results appropriately. Unfortunately, this point is not always clear in the literature. Instances can be found in which the width of the diagonal contour is taken as being proportional to the relaxation time (Ruta *et al.*, 2013 ▸; Bikondoa *et al.*, 2013 ▸) – *i.e.* the ACS-1-TCF convention is used – and where quantitative values of the relaxation time and the stretching parameter at different sample ages are reported. However, it would have been more natural to report quantitative values obtained following the CCS-1-TCF convention, as this is the one habitually used in glassy systems theory (Wolynes & Lubchenko, 2012 ▸). But because the ageing is so slow in the systems studied by Ruta *et al.* (2013 ▸) and Bikondoa *et al.* (2013 ▸), the ACS- and CCS-1-TCFs are essentially equivalent. In other work (Müller *et al.*, 2011 ▸), it is unclear if the 1-TCFs extracted from a 2-TCF that has sharp-cut division due to avalanche dynamics follow the CCS or the ACS convention. The example of the step function presented here in §5.1, suggests that the CCS-1-TCFs would be more suitable to analyse avalanche-type dynamics, and this may have been the procedure followed by Müller *et al.* (2011 ▸). But the reference provided by Müller *et al.* (2011 ▸) to explain how the 1-TCF has been calculated corresponds to work where the ACS-1-TCF was used (Malik *et al.*, 1998 ▸). Which reference system has been used by Shinohara *et al.* (2015 ▸) to extract 1-TCFs from 2-TCFs is not clear either. As there are different possible ways to extract 1-TCFs from 2-TCFs, it is important to explain precisely how the analysis has been carried out.

Non-equilibrium systems are arguably the most interesting cases to use the 2-TCF. Equilibrium systems are time translation invariant (Forster, 1995 ▸) but time symmetry is not retained in non-equilibrium systems. This symmetry break is reflected in the CCS-1-TCFs, but ACS-1-TCFs keep the symmetry for both equilibrium and non-equilibrium processes. On this basis, the CCS convention seems more convenient for the analysis of dynamic processes on non-equilibrium systems. But this does not preclude using the ACS convention if the theoretical analysis justifies it, as was done by Brown *et al.* (1997 ▸) and in subsequent work on the non-equilibrium dynamics of ordering systems and first-order transitions (see the references in the *Introduction* [Sec sec1]). Notwithstanding, we remark that for equilibrium systems both coordinate systems lead to the same result and that for systems in quasi-equilibrium the quantitative differences may be minor. The 2-TCFs could certainly be analysed using other slicing methods if the dynamics under study and their physical interpretation require it. A generic procedure to extract one-time correlation functions from multi-time correlation functions is presented in Appendix *A* [App appa].

## 7. Alternative representation of the two-time correlation function   

**Figure 8:**
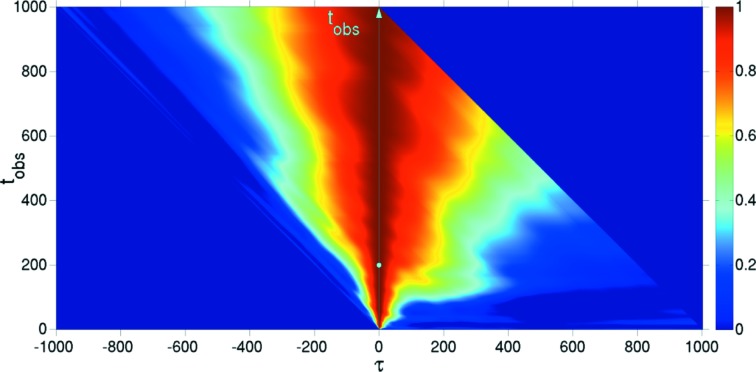
Alternative representation of the 2-TCF of Fig. 6 ▸. The observation time is denoted by the cyan arrow. The colour scale indicates the correlation.

We propose an alternative way to display graphically the 2-TCF in a way that the CCS coordinate system is more apparent. The 2-TCF elements are plotted according to the following matrix: 
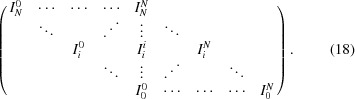
For a generic matrix term 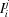 in equation (18), the observation time is 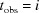 and the delay time 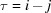. Graphically representing equation (18), the observation and delay times are along the vertical and horizontal axes, respectively (see Fig. 8 ▸). Negative/positive delay times correspond to going backward/forward in time. One advantage of this representation is that the 1-TCFs at different observation times are visualized more easily as horizontal lines. The autocorrelation function is obtained by averaging the rows instead of having to average diagonals. It also shows that with increasing sample age there are fewer terms for each of the 1-TCFs. In this graphical representation, the skewness and kurtosis of the peak at 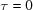 could be used to quantify the degree of departure from equilibrium and the correlation times. This assertion should still be cautioned: further theoretical developments are needed to verify if indeed the skewness and kurtoisis can meaningfully be related to the deviation from equilibrium, but the idea looks attractive.

## 8. Summary   

We have compared two coordinate systems that are used to analyse two-time correlation functions and extract one-time correlation functions from them. We have shown that taking one-time correlation functions along rows or columns (CCS-1-TCFs) is more compatible with the way autocorrelation functions are generally calculated and theoretical results reported. In certain cases, these CCS-1-TCFs are more consistent physically and do not present causality problems. Importantly, the CCS-1-TCFs are not necessarily symmetric by construction and thus a lack of time symmetry indicates that the system is not stationary. For non-equilibrium systems, the correlation and delay times that are obtained with this coordinate system differ from the ones that are obtained using the convention introduced by Brown *et al.* (1997 ▸) (ACS-1-TCFs). A new graphical representation of the 2-TCFs has been introduced, where the observation time is represented along the vertical axis and the delay time along the horizontal.

## Figures and Tables

**Figure 9:**
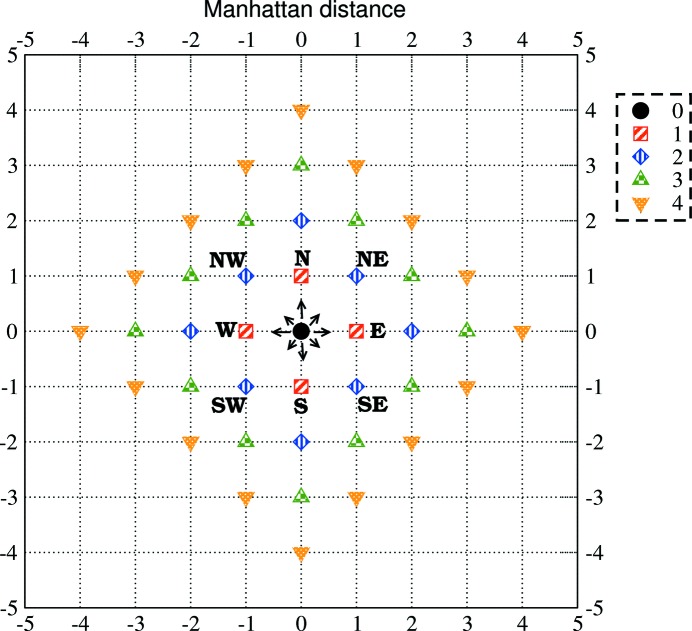
Manhattan distances on a two-dimensional grid. The Manhattan distances from point (0, 0) equal to 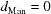, 1, 2, 3, 4 are represented by a circle, squares, diamonds, up triangles and down triangles, respectively. The tick indexes of axes *X* and *Y* indicate the index difference with respect to the point (0, 0).

**Figure 10:**
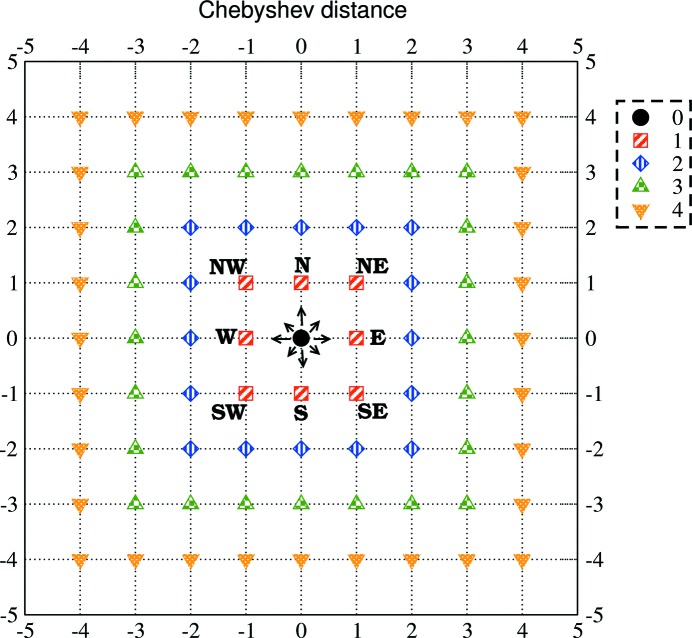
Chebyshev distances on a two-dimensional grid. The Chebybshev distances from point (0, 0) equal to 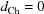, 1, 2, 3, 4 are represented by a circle, squares, diamonds, up triangles and down triangles, respectively. The tick indexes of axes *X* and *Y* indicate the index difference with respect to the point (0, 0).

**Table 1:** Terms at different delay times τ that are averaged when calculating the autocorrelation function [equation (8)]

τ	Terms	Number of terms
0	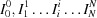	(*N* + 1)
1	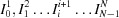	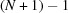
2	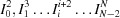	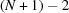
3	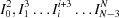	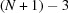
τ	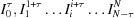	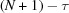
*N*	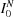	1

**Table 2:** Terms at different delay times extracted from the 2-TCF, following the conventions introduced in §§3.1 (CCS) and 3.2 (ACS) For a delay time τ to be accessible, the row and column numbers have to fulfil the inequalities specified above.

	Terms	
τ	CCS	ACS
0	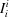	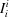
1	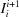	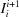
2	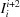	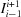
3	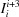	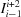
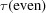	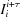	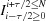
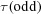	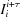	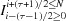

## Footnotes

1 The Siegert relation is valid if the coherence volume contains a large number of independent scatterers. In that case the central limit theorem conditions are met (Goodman, 1985 ▸). The total scattered field, which is the sum of many independent scatterers, is a random variable with a Gaussian probability distribution and the time correlation function of the intensity can be factorized to obtain the Siegert relation (Pusey, 2002 ▸).

2 The order in which the time and ensemble averages are performed can be very important. For example, information about the ergodicity of the system may be lost if the time average is done before the ensemble average (Pusey & Van Megen, 1989 ▸), but when using area detectors the time average should be done first to preserve the speckle visibility and to be able to extract measurement errors directly from azimuthal variations of  (Lumma *et al.*, 2000 ▸). However, multi-speckle dynamic light scattering (DLS)/PCS analysis is often done by performing the ensemble average first (Cipelletti & Weitz, 1999 ▸; Chushkin *et al.*, 2012 ▸). Thus, the choice of the top or bottom formula between the braces in equation (1) depends on the case under study. This aspect is beyond the scope of this manuscript. For more details, see the aforementioned references.

3 For the purposes of this paper, we will ignore the dependence of the intensity fluctuations on the scattering vector  and, to simplify the notation, we will not consider the normalization terms. See for example Chushkin *et al.* (2012 ▸), Madsen *et al.* (2015 ▸), Möller *et al.* (2016 ▸) and references therein for technical details about the measurement of intensity fluctuations and data processing.

4 In the usual matrix representation, the lowest row and column term is at the top left corner. Here, we represent the matrix setting the term with the lowest row and column index at the bottom left corner as the 2-TCF in XPCS is generally represented graphically in this manner.

5 In the following, we consider that using the CCS the 1-TCF is extracted along the rows. If it were extracted along the columns, the result would be equivalent owing to the symmetry of the 2-TCF matrix along the  diagonal.

## Appendix A Geometric description of multi-time correlation functions

We show here that multi-time (equivalently, multi-point) correlation functions can be conveniently expressed in terms of the formalism of metric spaces. Correlation functions of lower order are obtained using an adequate metric and defining a geometric trajectory in the multidimensional space. We describe how to construct generic α-time correlation functions from operations between *N*-tuples and how one-time correlation functions can be extracted from them. We pay special attention to the  case and the physical interpretation of the possible trajectories. We restrict ourselves to the correlation between only one variable. The generalization to correlations between different variables (cross correlations) is straightforward. A comprehensive discussion of arbitrary-order correlation functions using a tensor formalism, with special emphasis on coherence properties, is given by Mandel & Wolf (1995 ▸).

Let the tuple  be a set of measurements of the variable  made at times . Thus, the tuple indexes  are related to the time the measurement was done. The time difference (or temporal distance) between measurements is . Using the Cartesian product, we build an α-dimensional array

where α is the order of the correlation function we want to obtain. Each element of the array  is a tuple with α elements.

For each term in the array , we define the function       , where .  is the product of the elements of each α-tuple and yields the correlation function of order α. From , to extract an th-order correlation function we need to select a -dimensional subset of . Here, we sketch a method to obtain one-time correlation functions from an th-order correlation function.

First, we need to use a metric that defines the distance between the elements in the  set. The set and the metric define a ‘metric space’ (Reed & Barry, 1980 ▸). To extract a one-time correlation function from  we define a trajectory  on , . Starting from a point , the trajectory is chosen such that it joins points that are at consecutively larger distances in the  grid. The distance depends on the metric used.

The trajectories starting from a point   can be generically described as a set of points at successive *r* distances from *P*:

where  is the metric. As explained above, for a generic element , the indexes indicate which element of the tuple of measurements  are being multiplied when the function  is calculated, and are related to the time when the elements were measured. The Manhattan metric gives the distance between two elements in  as the sum of the absolute differences between their indexes:

where  and  are two generic points in . The Manhattan distance corresponds to the case  of the  norm (Deza & Deza, 2014 ▸):

The Manhattan distance is the equivalent of the delay time. In an  grid, there are many different ways (‘trajectories’) to join points at monotonically increasing Manhattan distances. In general, the one-time correlation functions along different trajectories starting at a point *P* will not be equivalent. We analyse in the following the trajectories on .

### A1. Trajectories in a two-time correlation function

A 2-TCF can be represented by a two-dimensional grid or matrix (see §2.2). The Moore neighbourhood of a point in a two-dimensional grid is the set of points surrounding it (Deza & Deza, 2014 ▸). If we denote the surrounding points using four cardinal (N, E, S, W) and four intercardinal points (NE, SE, SW, NW), the equal-time diagonal (*i.e.* terms of the form ) goes from the SW corner to the NE one (see Fig. 9 ▸). The allowed trajectories following one-unit step sizes of the Manhattan distance have individual steps going only along any of the four cardinal directions. With the Manhattan metric, trajectories along the intercardinal directions are obtained as staircase paths.  is symmetric by definition upon index swapping (*i.e.* ), so we restrict ourselves to trajectories that remain in only one part of , under the equal-time diagonal. Under there requisites, the most relevant trajectories, or at least those with a clear physical interpretation, are the trajectories starting at an equal-time point  and which go only eastwards (E), southwards (S) or south-eastwards (SE):

E. The eastwards trajectory mixes the event (measurement) at point *P* with measurements done at later times. This trajectory is equivalent to the usual autocorrelation function [equation (8)] except that there is no average between the trajectories that start at every point of the equal-time diagonal. The pair terms in the trajectory are of the form , where τ is given by the Manhattan distance. Averaging all the E trajectories for every point *P* on the equal-time diagonal, one obtains the usual autocorrelation function.

S. The southward trajectory relates the event (measurement) at point *P* with measurements done at earlier times. Thus, it can be interpreted as a correlation function where the delay time goes backwards in time. The terms in the trajectory are of the form . The S trajectory of any point *P* is the same as the W trajectory. Averaging all the S trajectories for every point *P* on the equal-time diagonal, one obtains the usual autocorrelation function.

SE. Using the Manhattan distance, the SE trajectory can only be obtained following a staircase-like trajectory. Depending on the choice of the term at a Manhattan distance equal to 1, the starting point *P* will be at the bottom or the top of the stair riser. In the SE trajectory, the event at time *P* only appears in the term at Manhattan distances 0 and 1. Terms at  relate events that happen before and after the event at *P*. The terms are of the form .

E, S or purely SE trajectories can be obtained using a Chebyshev metric instead of the Manhattan one as the selecting rule for the terms along a trajectory. The Moore neighbourhood of a point *P* is the set of points that are at a Chebyshev distance equal to 1. The Chebyshev metric corresponds to the  case of the  metric [equation (22)] and is defined as follows:

Two points in a grid at distance  can be joined by a unit displacement along any of the cardinal or intercardinal directions, *i.e.* by the movement of the king on a chessboard (see Fig. 10 ▸). The Chebyshev distance is also called the ‘chessboard’ or ‘king-move’ metric (Deza & Deza, 2014 ▸). A 1-TCF extracted from an SE trajectory starting at a point *P* of the equal-time diagonal and joining points at increasing Chebyshev distances is composed of terms arising from the multiplication of two events that happen at delay times  and , respectively (see Fig. 10 ▸).

There is an important difference between the 1-TCFs obtained using a Manhattan or a Chebyshev metric. The 1-TCFs obtained with a Manhattan metric always relate events that are at a unit delay time, whatever the direction of the steps is. However, using the Chebyshev metric, the delay time between the events that are related depends on the direction chosen. For 1-TCFs along only E or S (or staircase trajectories), the delay time is always 1. Along diagonals, the delay time between the related events is 2. That is, for a step with a Chebyshev distance equal to 1, the time step can in fact be 1 or 2.

It is clear that, depending on the metric used and the trajectories chosen, many different 1-TCFs can be constructed, which, in general, will not be equivalent. Other common metrics (for example, the Euclidean, which corresponds to the  with  case, and coincides with the Manhattan one if ) can yield completely different 1-TCFs from the Manhattan or Chebyshev metrics. In this particular case of time-correlation functions, the Manhattan norm yields a clear physical picture for any dimensions, because  always relates events that are separated by the same delay time, independently of the direction chosen in the α-multidimensional space. For point (position) correlation functions obtained from measurements on a plane, Euclidean metrics would be better suited. The physics of the problem treated will determine which metric should be used.
